# Design and dynamic analysis of jumping wheel-legged robot in complex terrain environment

**DOI:** 10.3389/fnbot.2022.1066714

**Published:** 2022-12-02

**Authors:** Tiezheng Guo, Jinhui Liu, Haonan Liang, Yitong Zhang, Wei Chen, Ximing Xia, Meiqing Wang, Zhiming Wang

**Affiliations:** ^1^Industrial Center, Nanjing Institute of Technology, Nanjing, Jiangsu, China; ^2^Direct Drive Technology Ltd., Dongguan, Guangdong, China; ^3^Key Laboratory of Crop Harvesting Equipment Technology of Zhejiang Province, Jinhua, Zhejiang, China

**Keywords:** complex terrain environment, wheel-legged robot, dynamic analysis, adaptive obstacle crossing, motion analysis

## Abstract

Wheel-legged robots have fast and stable motion characteristics on flat roads, but there are the problems of poor balance ability and low movement level in special terrains such as rough roads. In this paper, a new type of wheel-legged robot with parallel four-bar mechanism is proposed, and the linear quadratic regulator (LQR) controller and fuzzy proportion differentiation (PD) jumping controller are designed and developed to achieve stable motion so that the robot has the ability to jump over obstacles and adapt to rough terrain. The amount of energy released by the parallel four-bar linkage mechanism changes with the change of the link angle, and the height of the jump trajectory changes accordingly, which improves the robot’s ability to overcome obstacles facing vertical obstacles. Simulations and real scene tests are performed in different terrain environments to verify obstacle crossing capabilities. The simulation results show that, in the pothole terrain, the maximum height error of the two hip joint motors is 2 mm for the obstacle surmounting method of the adaptive retractable wheel-legs; in the process of single leg obstacle surmounting, the maximum height error of the hip joint motors is only 6.6 mm. The comparison of simulation data and real scene experimental results shows that the robot has better robustness in moving under complex terrains.

## Introduction

With the continuous development of robot technology, the application scope of mobile robots is constantly expanding, and the diversification of application scenarios leads to the increase of robots facing complex terrain environments (Zhang et al., 2014; Gao et al., 2020). Wheeled robots have the advantages of high efficiency and high energy utilization on flat roads (Wu, 2020; Xin et al., 2020), but they have poor adaptability to complex terrain. When the height of obstacles is greater than the radius of the wheels, they cannot effectively cross obstacles (Ding and Zhang, 2022). Legged robots have excellent adaptability when moving on the uneven and rough roads, but slow moving speed and low movement energy efficiency have always been technical problems that are difficult to break through (Xin et al., 2019; Xin and Vijayakumar, 2020). To solve this problem, the researchers turned their attention to wheel-legged robots. Wheel-legged robots combine the advantages of wheeled robots and legged robots. Double wheels can maximize energy utilization efficiency and maneuverability, and the leg structure makes the robot more adaptable to complex terrain environments.

At present, scholars have carried out a lot of research on the stability and high obstacle crossing ability of wheel-legged robots in unstructured terrain and have achieved a series of results: Liu et al. (2019, 2022) and Zhang et al. (2019) proposed a bipedal wheeled robot SR600 for logistics in scenarios such as distribution and home services, it can change height while maintaining dynamic balance. The size design of the human body can make it better interact with people. Kim et al. (2014) developed the Wheel Transformer, a variable-diameter wheel-legged robot. When encountering an obstacle, the wheels are transformed into two three-legged wheels to complete the action of crossing over the obstacle. It can overcome obstacles that 3.25 times higher than the wheel radius, but there is also the problem of low efficiency of crossing obstacles. In nature, animals jump over obstacles and avoid enemies attack by jumping (Fei et al., 2012; Cheng, 2021). Inspired by this, the bionic jumping theory was applied to the wheel-legged robot, and the jumping obstacle was realized by the wheel-legged robot (Zhuang et al., 2021; Hao et al., 2022). Chen et al. (2021) studied the jumping of a bipedal wheel-legged robot, proposed a W-SLIP model to characterize the jumping process dynamics, and verified the robot’s jumping performance through V-REP simulation. Bipedal wheel-legged robot Ascento produced by ETH Zurich that adopted a compact design structure and can jump over obstacles while keeping the robot flexible and compact (Klemm et al., 2019; Klemm et al., 2020). The quadruped wheel-legged robot ANYmal (Bjelonic et al., 2019) of ETH Zurich also reflected the advantages of the combination of legged robots and wheeled robots to a large extent. The typical wheel-legged robot Handle developed by Boston Dynamics Ltd (2017). Achieved self-balancing through a dynamic control center, and used a hydraulic drive to jump to a height of 1.2 m (Zhang et al., 2018). At present, wheel-legged robots are still mainly used in simple application scenarios such as logistics handling, while wheel-legged robots suitable for complex terrain environments have been rarely reported.

Aiming at the problems that the wheel-legged robot is not stable in the complex terrain environment, a jumping wheel-legged robot is proposed in this paper, and an adaptive retractable wheel-leg mechanism is designed to keep the body parallel to the ground to achieve smooth obstacle-surmounting. The main motion forms of the robot include wheel and wheel-leg movement modes, and the modes are switched by the rotation of the hip motor. The wheel movement mode is used on flat terrain, and the wheel-leg movement mode is used on obstacles and rough terrain to improve energy utilization efficiency. In the wheel-leg movement mode, the robot cannot only use the wheels to move quickly and stably, but also jump over obstacles through the expansion and contraction of the wheel-leg linkage mechanism. The paper is structured as follows: In Section “Kinematics and dynamics analysis,” the robot is introduced and the kinematics and dynamics modeling, including the self-balancing dynamics model and the jumping dynamics model. In Section “Analysis of obstacle crossing conditions,” the wheeled overcoming obstacles and jumping over obstacles are analyzed respectively, and the conditions for overcoming the obstacles are obtained. The control system is presented in Section “Robot Control System,” divided into self-balancing and jump control. Section “Simulation and experiment” conducts simulation tests on robot crossing obstacles in different terrains, and conducts actual test verifications. The paper is concluded in the last section.

## Kinematics and dynamics analysis

**FIGURE 1:**
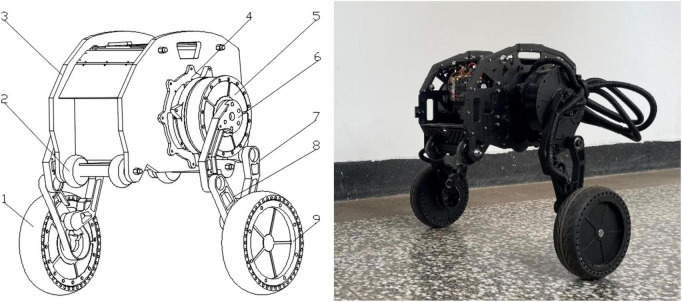
Schematic diagram of the robot structure. 1. Right wheel (driving wheel); 2. Universal wheel; 3. Body; 4. Waist motor; 5. Hip motor; 6. Connecting rod 1; 7. Connecting rod 2; 8. Connecting rod 3; 9. Left wheel (driving wheel).

**FIGURE 2:**
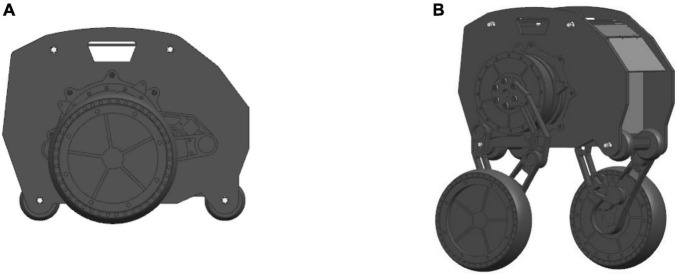
Schematic diagram of robot motion posture: **(A)** Wheel movement mode; **(B)** Wheel-leg movement mode.

The overall structure design of the robot is shown in Figure 1, which consists of the body, six motors, two linkage mechanisms, two pairs of universal wheels and two wheels. The two waist motors (including No. 4 in Figure 1) are hidden inside the body, and two motors are installed in the left and right wheels to drive the wheels to move. The waist motors are used to adjust the pitch angle of the body, and the hip motors are used to control the robot to change the height of the body and realize the jumping function. The hip motor rotates, and under the action of gravity, the connection between the Connecting rod 2 and the hip motor rotates around the rotation axis of the hip joint to realize the expansion and contraction of the leg linkage mechanism. The height of the body is raised and lowered through the expansion and contraction of the linkage mechanisms, and the wheel-leg linkage mechanisms contract and stretch in a short time, completing the accumulation and release of the energy required for jumping, and the robot realizes the jumping action. The wheel movement mode and the wheel-leg movement mode are shown in Figures 2A,B below, respectively.

### Kinematic modeling

**FIGURE 3:**
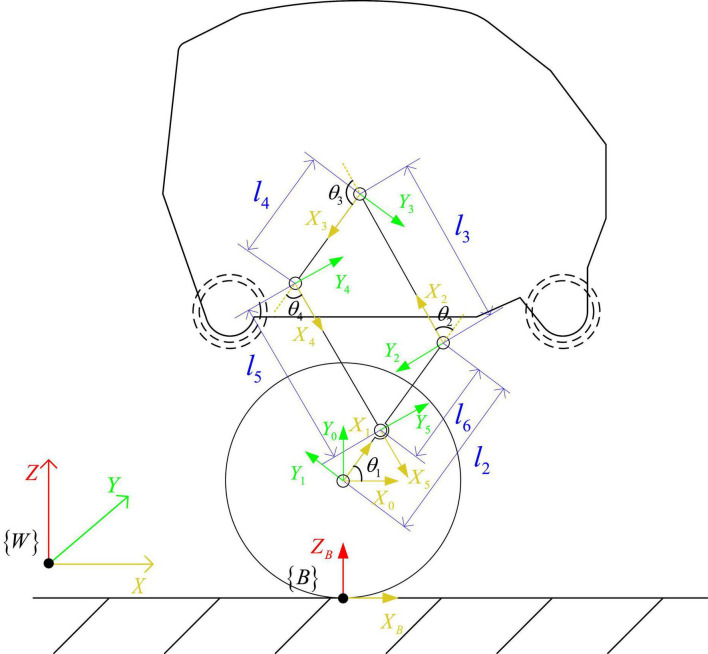
Kinematics model of robot.

As shown in Figure 3, the kinematic model of the standing posture of the wheel-legged robot is established. {*W*} is the world coordinate system, the *Z* axis is vertically upward, the *X* axis is perpendicular to the *Z* axis and points to the right end, and the *Y* axis direction is determined according to the right-hand rule. In order to simplify the kinematics problem, the base coordinate system {*B*} is established at the contact point between the wheel and the ground, the direction is parallel to the world coordinate system, and the coordinate system {0} is established at the center of the wheel. The coordinate system {1} is established at the connection between the Connecting rod 3 and the wheel, the coordinate system {2} is established at the connection between the Connecting rod 1 and the Connecting rod 3, and the coordinate system is established at the position shown in the Figure 3 in turn. The *Z*_*i*_(*i* = 1∼5) axis of the link is along the joint. The positive direction of the axis is placed perpendicular to the surface of the paper, the positive direction of the *X_i_* axis points to the common perpendicular of the *i* axis and the *i+*1 axis, and the direction of the *Y_i_* axis is determined by the right-hand rule.

In the kinematic model of Figure 3, *a*_*i–1*_ is the length of the connecting rod, α_*i*−1_ is the rotation angle of the connecting rod, *d_i_* is the offset distance of the connecting rod, θ_*i*_ is the joint angle, *l_i_* is the distance between the origin of the coordinate system {*i*−1} and the origin of the coordinate system {*i*} in the Z-X plane. where *i* = 1∼5 is the rotational joint of the robot, *d*_1_ = 0.026*m*, *d*_2_ = 0.021*m*, *d*_3_ = 0.016*m*, *d*_5_ = 0.016*m*. *l*_2_ = 0.14*m*, *l*_3_ = 0.14*m*, *l*_4_ = 0.09*m*, *l*_5_ = 0.14*m*, *l*_6_ = 0.09*m*, the robot wheel radius *r* = 0.095*m*.

According to the established kinematics model, the forward kinematics is solved, and the connecting rod transformation matrix is obtained as:


(1) T10=[C1-S100S1C10000100001] ,T20=[C12-S120C1⁢l2S12C120S1⁢l2001-d1-d20001]

(2) T30=[C123-S1230C1⁢l2+C12⁢l3S123C1230S1⁢l2+S12⁢l3001-d1-d2-d30001] ,T40=[C1234-S12340C1⁢l2+C12⁢l3+C123⁢l4S1234C12340S1⁢l2+S12⁢l3+S123⁢l4001-d1-d2-d30001]

(3) T50=[C1234-S12340C1⁢l2+C12⁢l3+C123⁢l4+C1234⁢l5S1234C12340S1⁢l2+S12⁢l3+S123⁢l4+S1234⁢l5001d5-d1-d2-d30001]

Where, *C*_*1234*_ is the meaning of cos(θ_1_ + θ_2_ + θ_3_ + θ_4_), *S*_*1234*_ is the meaning of sin(θ1+θ2+θ3+θ)4.

The robot is a parallelogram linkage mechanism, and the robot body is expected to be parallel to the ground while maintaining balance, so the constraint equation is attached:


(4) $$\theta_{2}=\theta_{4}=\pi -\theta_{3},\theta_{1}=\frac{1}{2}\,\theta_{3}$$

Use UG software to analyze the position of the center of mass of each rod and the body of the robot, and obtain the coordinates of the center of mass *c*1 of the Connecting rod 3, the center of mass *c*2 of the Connecting rod 1, the center of mass *c*3 of the body, and the center of mass *c*4 of the Connecting rod 2 relative to the *X*-*Y* plane of the {1}, {2}, {3}, {4} coordinate system:


(5) {Xc⁢11=0.0545Yc⁢11=0 ,{Xc⁢22=0.0802Yc⁢22=-0.0128 ,{Xc⁢33=-0.0219Yc⁢33=-0.0235 ,{Xc⁢44=0.0714Yc⁢44=-0.0152

The angle between the line connecting the center of mass *c*2 and the origin of the coordinate system {2} and the positive direction of the *X_2_* axis is φ_2_ = *atan*2(^2^*Y*_*c*2_,^2^*X*_*c*2_), the angle between the line of the center of mass *c*3 and the origin of the coordinate system of {3} and the positive direction of the *X_3_* axis is φ_3_ = *atan*2(^3^*Y*_*c*3_,^3^*X*_*c*3_), the angle between the line connecting the center of mass c4 and the origin of the coordinate system of {4} and the positive direction of the *X_4_* axis is φ_4_ = *atan*2(^4^*Y*_*c*4_,^4^*X*_*c*4_), the center of mass of the hip motor *c*5 is at the center of rotation. Let the lengths of the center of mass *c*1, *c*2, *c*3, and *c*4 from the origin of the {1}, {2}, {3}, and {4} coordinate systems be *l*_*c1*_, *l*_*c2*_, *l*_*c3*_, and *l*_*c4*_, respectively. Then the position of the center of mass of each rod *ci* relative to the world coordinate system is (*X*_*ci*_, *Z*_*ci*_), and the velocity is $\sqrt{\dot{X_{c\,i}^{2}}+\dot{Z_{c\,i}^{2}}}$, *i* = 1∼5.

### Dynamic modeling

#### Self-balancing dynamic modeling

**FIGURE 4:**
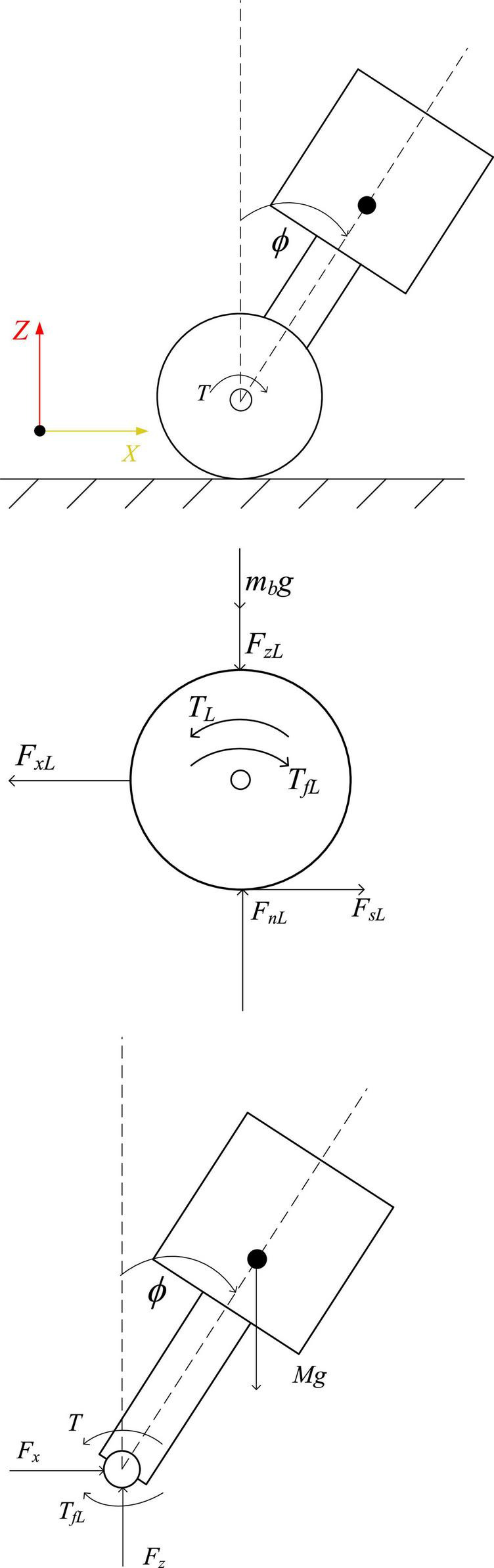
Force diagram of equivalent model.


When the robot maintains a standing posture, the leg linkage mechanism is kept fixed by locking the hip joint motor. At this time, the robot can be equivalent to a two-wheeled self-balancing robot, as shown in Figure 4. The center of mass is located above the wheel axis of the robot, and the pose of the robot in the world coordinate system is [*x*_*b*_,*y*_*b*_,*z*_*b*_,α]^*T*^, where the position coordinate of the midpoint of the axis of the driving wheels of the robot is [*x*_*b*_,*y*_*b*_,*z*_*b*_ + *r*], α is the heading angle of the robot, the distance between the centers of the two wheels is *D*, and the radius of the wheel is *r*, the angles that the left and right wheels have turned are θ_*L*_,θ_*R*_, and the displacements of the left and right wheels are *x*_*L*_,*x*_*R*_, respectively. Assume that the body mass of the simplified robot is *M*, the length of the body is *L*, the moment of inertia of the body around the *Y* axis is *I_b_*, and the position coordinate of the center of mass of the body is [*x*, 0,*z*]. The moment of inertia of each connecting rod at the center of mass is *I*_*ci*_, which is obtained with the assistance of Adams simulation software. The tilt angle and body length of the equivalent model are:

(6) $$\phi =\arctan\left(\frac{x}{z-r}\right)\;,L=\sqrt{x^{2}+{\left(z-r\right)}^{2}}$$

Taking the left wheel of the robot as an example to analyze the force on the wheel and the body, the balance formula of the force and moment of the body and the wheel can be obtained:


(7) $$\left\{ \begin{array}{c} F_{s\,L}-F_{x\,L}=m\,{\ddot{x}}_{L}\\ F_{n\,L}-F_{z\,L}=m_{b}\,g \end{array}\right.\;,\left\{ \begin{array}{c} T_{L}-r\,F_{s\,L}-T_{f\,L}=I_{W}\,{\ddot{\theta }}_{L}\\ T_{f\,L}=b\,\left({\dot{\theta }}_{L}-\dot{\phi }\right) \end{array}\right.$$

According to the relationship between the displacement of the midpoint of the line connecting the centers of the two wheels and the wheel rotation, the equation can be obtained:


(8) $$\left(T_{L}+T_{R}\right)-\;r\;\left(F_{s\,R}+F_{s\,L}\right)-\;b\;\left({\dot{\theta }}_{L}+{\dot{\theta }}_{R}\right)+2\,b\,\dot{\phi }-I_{w}\,\left({\ddot{\theta }}_{L}+{\ddot{\theta }}_{R}\right)=0$$

According to the balance relationship between force and moment, follows is got:


(9) $$\begin{array}{c} \left(T_{L}+T_{R}\right)+M\,L\,\cos\phi \,\left({\ddot{x}}_{b}+\ddot{\phi }\,L\,\cos\phi -{\dot{\phi }}^{2}\,L\,\sin\phi \right)+\\ ML\sin\phi (-g+\ddot{\phi }L\sin\phi +\\ {\dot{\phi }}^{2}L\cos\phi )-b\;({\dot{\theta }}_{L}+{\dot{\theta }}_{R})+2b\dot{\phi }=I{}_{b}\ddot{\phi } \end{array}$$

Among them, assuming that there is no slippage between the wheels and the ground, then *x*_*L*_ = *r*θ_*L*_, the relationship between the displacement of the midpoint of the line connecting the centers of the two wheels and the left and right wheel angles is: *x*_*b*_=1/2(θ_*L*_ + θ_*R*_). *I_w_* is the moment of inertia of the wheel, *F_x_* is the interaction force between the wheel and the body in the *X*-axis direction, *F_z_* is the interaction force between the wheel and the body in the *Y*-axis direction, *T_L_* and *T_R_* are the output torques of the left and right wheel motors, respectively, *T*_*fL*_ and *T*_*fR*_ are the friction torque, *b* is the friction coefficient between the wheel and the rod, the support force between the wheel and the ground is *F_n_*, and the friction force is *F_s_*.

**FIGURE 5:**
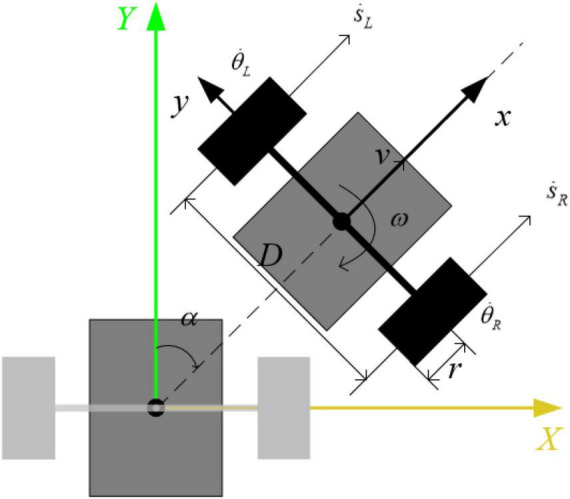
Top view of the robot.


The top view of the robot is shown in Figure 5, the ${\dot{s}}_{L}$ and ${\dot{s}}_{R}$ are the speeds of the left and right wheels in the X direction, respectively. Assuming that the moment of inertia of the robot around the vertical direction is *I_t_*, the robot realizes differential turning when there is a differential speed between the two wheels. The turning dynamics equation is:

(10) $$\left(\frac{D\,I_{w}}{r}+m\,D\,r+\frac{2\,I_{t}\,r}{D}\right)\,\ddot{\alpha }=T_{L}-T_{R}$$

The dynamic equations of the robot in the self-balancing mode can be sorted out as:


(11) $$\left\{ \begin{array}{c} T_{L}+T_{R}-{\ddot{x}}_{b}\,\left(2\,m_{b}\,r+M\,r+I_{w}\,\frac{2}{r}\right)-\\ M\,r\,\left(\ddot{\phi }\,L\,\cos\phi -{\dot{\phi }}^{2}\,\sin\phi \right)-2\,b\,\left(\frac{{\bar{x}}_{b}}{r}+\dot{\phi }\right)=0\\ {\ddot{x}}_{b}\,M\,L\,\cos\phi -M\,g\,L\,\sin\phi +\ddot{\phi }\,\left(M\,L^{2}-I_{b}\right)-\\ \frac{2\,b}{r}\,{\dot{x}}_{b}+2\,b\,\dot{\phi }+T_{L}+T_{R}=0\\ \left(\frac{D\,I_{w}}{r}+m_{b}\,D\,r+\frac{2\,I_{t}\,r}{D}\right)\,\ddot{\alpha }=T_{L}-T_{R} \end{array}\right.$$

Organize the dynamic equations into non-linear dynamic equations in state-space form:


(12) $$\left\{ \begin{array}{c} \ddot{\phi }=\frac{\begin{array}{c} \left(a_{21}\,\cos\phi -a_{11}\right)\,\left(T_{L}+T_{R}\right)+\\ \left(a_{11}\,a_{24}-a_{15}\,a_{21}\,\cos\phi \right)\,{\dot{x}}_{b}-\left(a_{14}\,a_{21}+a_{11}\,a_{25}\right)\,\dot{\phi }\\ +a_{13}\,a_{21}\,{\dot{\phi }}^{2}\,\sin\phi \,\cos\phi +a_{11}\,a_{22}\,\sin\phi \end{array}}{a_{11}\,a_{23}+a_{12}\,a_{21}\,\cos^{2}\phi }\\ {\ddot{x}}_{b}=\frac{\begin{array}{c} -\left(a_{23}+a_{12}\,\cos\phi \right)\,\left(T_{L}+T_{R}\right)-a_{13}\,a_{23}\,{\dot{\phi }}^{2}\,\sin\phi +\\ \left(a_{14}\,a_{23}-a_{12}\,a_{25}\,\cos\phi \right)\,\dot{\phi }\\ +\left(a_{15}\,a_{23}+a_{12}\,a_{24}\,\cos\phi \right)\,{\dot{x}}_{b}+a_{12}\,a_{22}\,\sin\phi \,\cos\phi \end{array}}{a_{11}\,a_{23}+a_{12}\,a_{21}\,\cos^{2}\phi }\\ \ddot{\alpha }=\frac{T_{L}-T_{R}}{a_{31}} \end{array}\right.$$

Where, $a_{11}=2\,m_{b}\,r+M\,r+\frac{2\,I_{w}}{r}$, *a*_12_ = *MLr*, *a*_13_ = *Mr*, *a*_14_ = 2*b*, $a_{15}=\frac{2\,b}{r}$, *a*_21_ = *ML*, *a*_22_ = *Mgl*, *a*_23_ = *ML*^2^−*I*_*b*_, $a_{24}=\frac{2\,b}{r}$, *a*_25_ = 2*b*, $a_{31}=\frac{D\,I_{w}}{r}+m_{b}\,D\,r+\frac{2\,I_{t}\,r}{D}$.

For the dynamic equation of the robot in the self-balancing mode, take $X={\left[x_{b},{\dot{x}}_{b},\phi ,\dot{\phi },\alpha ,\dot{\alpha }\right]}^{T}$ as the system state variable and *u* = [*T*_*L*_,*T*_*R*_]^*T*^ as the system input variable for linearization. Assuming that the robot is near the equilibrium position, there is ϕ ≈ 0, $\dot{\phi }\approx 0$, which is brought into the non-linear dynamic equation to obtain the linearization of the system. Equation of state:


(13) $$\left[\begin{array}{c} {\dot{x}}_{b}\\ {\ddot{x}}_{b}\\ \dot{\phi }\\ \ddot{\phi }\\ \dot{\alpha }\\ \ddot{\alpha } \end{array}\right]=[\begin{array}{cccc} 0 & 1 & 0 & \begin{array}{cc} 0 & \begin{array}{cc} 0 & 0 \end{array} \end{array}\\ 0 & A_{22} & A_{23} & \begin{array}{cc} 0 & \begin{array}{cc} 0 & 0 \end{array} \end{array}\\ 0 & 0 & 0 & \begin{array}{cc} 1 & \begin{array}{cc} 0 & 0 \end{array} \end{array}\\ \begin{array}{c} 0\\ 0\\ 0 \end{array} & \begin{array}{c} A_{42}\\ 0\\ 0 \end{array} & \begin{array}{c} A_{43}\\ 0\\ 0 \end{array} & \begin{array}{cc} \begin{array}{c} 0\\ 0\\ 0 \end{array} & \begin{array}{c} \begin{array}{cc} 0 & 0 \end{array}\\ \begin{array}{cc} 0 & 1 \end{array}\\ \begin{array}{cc} 0 & 0 \end{array} \end{array} \end{array} \end{array}]\;\,\left[\begin{array}{c} x_{b}\\ {\dot{x}}_{b}\\ \phi \\ \dot{\phi }\\ \alpha \\ \dot{\alpha } \end{array}\right]+[\begin{array}{cc} 0 & 0\\ B_{2} & B_{2}\\ 0 & 0\\ B_{4} & B_{4}\\ 0 & 0\\ B_{6} & -B_{6} \end{array}]\;\,\left[\begin{array}{c} T_{L}\\ T_{R} \end{array}\right]$$

Among them,


$$\begin{array}{c} \Delta =r^{2}\,\left(2\,M^{2}\,L^{2}+2\,m_{b}\,M\,L^{2}-2\,m_{b}\,I_{b}-M\,I_{b}\right)+2\,M\,L^{2}\,I_{w}-2\,I_{w}\,I_{b}\\ A_{22}=2\,\left(b\,M\,L^{2}-b\,I_{b}+M\,L\,r\,b\right)/\Delta \\ A_{23}=M^{2}\,g\,L^{2}\,r^{2}/\Delta \\ A_{42}=\left(4\,b\,r\,m_{b}+2\,M\,b\,r+4\,b\,I_{w}/r-2\,M\,b\,L\right)/\Delta \\ A_{43}=\left(2\,M\,m_{b}\,g\,L\,r^{2}+M^{2}\,g\,L\,r^{2}+2\,I_{w}\,M\,g\,L\right)/\Delta \\ B_{2}=\left(-M\,L^{2}\,r+I_{b}\,r-M\,L\,r^{2}\right)/\Delta \\ B_{4}=\left(-2\,m_{b}\,r^{2}-M\,r^{2}-2\,I_{w}+M\,L\,r\right)/\Delta \\ B_{6}=D\,r/\left(D^{2}\,I_{w}+m_{b}\,D^{2}\,r^{2}+2\,I_{t}\,r^{2}\right) \end{array}$$

The linear system is decoupled into a separate balance subsystem and steering subsystem, and the straight-running torque *T*_ϕ_ and the steering torque *T*_ω_ of the robot are, respectively, input. The relationship between the left and right wheel torques and *T*_ϕ_ and *T*_ω_ is expressed as a matrix:


(14) $$\left[\begin{array}{c} T_{L}\\ T_{R} \end{array}\right]=\left[\begin{array}{cc} 0.5 & 0.5\\ 0.5 & 0.5 \end{array}\right]\,\left[\begin{array}{c} T_{\phi }\\ T_{\omega } \end{array}\right]$$

Then the state space equations of the equilibrium system and the steering system are:


(15) $${\dot{X}}_{1}=A_{1}\,X_{1}+B_{1}\,T_{\phi },{\dot{X}}_{2}=A_{2}\,X_{2}+B_{2}\,T_{\omega }$$

Where,


(16) $$X_{1}=\left[\begin{array}{c} x_{b}\\ {\dot{x}}_{b}\\ \phi \\ \dot{\phi } \end{array}\right],A_{1}=\left[\begin{array}{cccc} 0 & 1 & 0 & 1\\ 0 & A_{22} & A_{23} & 0\\ 0 & 0 & 0 & 1\\ 0 & A_{42} & A_{43} & 0 \end{array}\right],B_{1}=\left[\begin{array}{c} 0\\ B_{2}\\ 0\\ B_{4} \end{array}\right]\;,\;X_{2}=\left[\begin{array}{c} \alpha \\ \dot{\alpha } \end{array}\right],A_{2}=\left[\begin{array}{cc} 0 & 1\\ 0 & 0 \end{array}\right],B_{2}=\left[\begin{array}{c} 0\\ B_{6} \end{array}\right].$$

#### Jump phase dynamic modeling

The inertia tensor of the robot is calculated by Adams software. The coordinate origin of the inertia tensor is the center of mass of each rod. The jumping action of the robot is analyzed on the *XOZ* plane of the base coordinate system. The rotation axis of the rod is the *Z* axis of the corresponding joint coordinate system. In order to simplify the calculation of the inertia tensor, let the coordinate system with the center of mass of each rod as the origin of the inertia tensor coincide with the three inertia axes of the rod, and according to the parallel shift axis theorem, the change of the rotation axis of the connecting rod only changes the size of the moment of inertia in the inertia tensor, so the inertia tensor can be easily calculated using Adams software.

In this paper, the Lagrangian equation is used to establish the dynamic model of the wheel-legged robot, and θ_3_ is used as the generalized coordinate, and the Lagrangian equation is applied to the process of the robot’s take-off phase. The kinetic energy *K* and potential energy *V* of the system are:


(17) $$\left\{ \begin{array}{c} K_{0}=\frac{1}{2}\,m_{b}\,{\dot{X}}_{B}^{2}\\ K_{1}=\frac{1}{2}\,m_{c\,1}\,\left({\dot{X}}_{c\,1}^{2}+{\dot{Z}}_{c\,1}^{2}\right)+\frac{1}{2}\,I_{\mathrm{c1}}\,{\dot{\theta }}_{1}^{2}\\ K_{2}=\frac{1}{2}\,m_{c\,2}\,\left({\dot{X}}_{c\,2}^{2}+{\dot{Z}}_{c\,2}^{2}\right)+\frac{1}{2}\,I_{\mathrm{c2}}\,{\left({\dot{\theta }}_{1}+{\dot{\theta }}_{2}\right)}^{2}\\ K_{3}=\frac{1}{2}\,m_{c\,3}\,\left({\dot{X}}_{c\,3}^{2}+{\dot{Z}}_{c\,3}^{2}\right)\\ K_{4}=\frac{1}{2}\,m_{c\,4}\,\left({\dot{X}}_{c\,4}^{2}+{\dot{Z}}_{c\,4}^{2}\right)+\frac{1}{2}\,I_{\mathrm{c4}}\,{\left({\dot{\theta }}_{1}+{\dot{\theta }}_{2}+{\dot{\theta }}_{3}+{\dot{\theta }}_{4}\right)}^{2}\\ K_{5}=\frac{1}{2}\,m_{c\,5}\,\left({\dot{X}}_{c\,5}^{2}+{\dot{Z}}_{c\,5}^{2}\right)+\frac{1}{2}\,I_{\mathrm{c5}}\,{\dot{\theta }}_{3}^{2} \end{array}\right.$$

(18) $$V=2\,m_{c\,1}\,g\,Z_{c\,1}+2\,m_{c\,2}\,g\,Z_{c\,2}+m_{c\,3}\,g\,Z_{c\,3}+2\,m_{c\,4}\,g\,Z_{c\,4}+2\,m_{c\,5}\,g\,Z_{c\,5}+2\,m_{b}\,g\,Z_{b}$$

(19) $$L=2\,K_{0}+2\,K_{1}+2\,K_{2}+K_{3}+2\,K_{4}+2\,K_{5}-V$$

Where, *L* is the Lagrangian, which represents the difference in value between the kinetic energy *K* and potential energy *V* of the robot.

The hip joint motor torque $\tau =\left[\frac{d}{d\,t}\,\left(\frac{\partial K}{\partial {\bar{\theta }}_{3}}\right)-\frac{\partial K}{\partial \theta_{3}}+\frac{\partial V}{\partial \theta_{3}}\right]/2$ is obtained, and the hip joint space dynamics state space equation of the robot is sorted out as:


(20) $$M\,\left(q\right)\,\ddot{q}+V\,\left(q,\dot{q}\right)+G\,\left(q\right)=\tau$$

Where, *M*(*q*) is the inertia matrix of the robot, $V\,\left(q,\dot{q}\right)$ is the centrifugal and Coriolis matrix, and *G*(*q*) is the gravity compensation vector.

## Analysis of obstacle crossing conditions

The robot’s ability to overcome obstacles is mainly affected by its own structure and road conditions. The wheel movement mode of the wheel-legged robot proposed in this paper is mainly used for smooth road movement, and the wheel-leg mode is used to pass the rough road with obstacles. In the following, the obstacle crossing analysis is carried out, respectively, for the cases that the obstacle height is lower than the wheel radius and the obstacle height is higher than the wheel radius in the wheel-leg mode.

### Wheeled obstacle crossing analysis

When the height of the obstacle is less than the radius of the wheel, the analysis is performed at the initial stage of the robot crossing the obstacle. At the beginning of obstacle crossing, keep the center of mass of the robot and the center of rotation of the wheel on the same vertical line, and perform force analysis on it.

**FIGURE 6:**
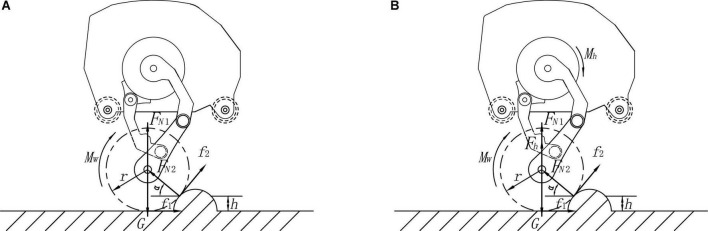
Wheel over obstacles in wheel-leg mode: **(A)** Cross obstacle on two legs; **(B)** Cross obstacle on one leg.


Figure 6 shows the robot cross over the obstacle in wheel-leg mode, at the beginning of obstacle crossing, the robot is balanced by the forces and moments between the ground and the wheels and between the obstacles and the wheels. The balance formula of the robot’s two legs over obstacle is shown in Equation (21):

(21) $$\left\{ \begin{array}{c} M-G\,r\,\cos\alpha +F_{N\,1}\,r\,\cos\alpha =0\\ F_{N\,2}\,\cos\alpha -f_{1}-f_{2}\,\sin\alpha =0\\ G-F_{N\,1}-F_{N\,2}\,\sin\alpha =0 \end{array}\right.$$

In Equation (21), *M_w_* is the torque of the wheel motor, *G* is its own gravity, *F*_*N1*_ is the support force of the ground facing the wheel, *F*_*N2*_ is the support force of the obstacle contact point to the wheel, *f_1_* is the friction force of the ground facing the wheel, and *f_2_* is the frictional force on the wheel at the contact point of the obstacle, α is the angle between the supporting force of the contact point between the wheel and the obstacle and the ground, *h* is the height between the contact point of the obstacle and the ground, and *r* is the radius of the wheel.

The balance formula of the robot’s one leg over obstacle is shown in Equation (22):


(22) $$\left\{ \begin{array}{c} M_{w}-G\,r\,\cos\alpha +F_{N\,1}\;r\;\cos\alpha +F_{h}\,r\,\cos\alpha =0\\ F_{N\,2}\;\cos\alpha -f_{1}-f_{2}\,\sin\alpha =0\\ G-F_{N\,1}-F_{N\,2}\;\sin\alpha -F_{h}=0 \end{array}\right.$$

In Equation (22), *M_h_* and *F_h_* are, respectively, the torque of the hip motor in the adaptive contraction state of the robot when crossing the obstacle with one leg, and the force transmitted by the torque of the hip motor to the wheel through the connecting rod of the wheel leg. The meanings of other parameters are consistent with Equation (21).

In Equations (21, 22), *f*_1_=μ*F*_*N*1_, *f*_2_=μ*F*_*N*2_, $\alpha =a\,r\,c\,s\,i\,n\,\frac{r-h}{r}$. When α > 0, the wheel can advance over the obstacle by its own rotational motion driven by the motor.

### Analysis of jumping over obstacles

In the analysis of the robot jumping over obstacles, due to the symmetric design of the robot, the center of mass in the process of movement can be kept only in the *X* and *Z* direction displacement in the world coordinate system, and the jumping process is analyzed according to the plane robot analysis method. Assume that the take-off phase time of the robot is [*t*_0_,*t*_1_) and the flight phase time is [*t*_1_,*t*_4_). If the robot wants to jump off the ground after energy storage, it needs to meet the following conditions:


(23) $$\left\{ \begin{array}{c} {\dot{Z}}_{c}>0\\ {\ddot{Z}}_{c}=-g \end{array}\right.$$

Therefore, the following conditions should be met in the take-off phase time [*t*_0_,*t*_1_):


(24) $$\left\{ \begin{array}{c} {\dot{X}}_{c}\,\left(t_{1}\right)={\dot{X}}_{c\,0}\geq 0\\ {\dot{Z}}_{c}\,\left(t_{1}\right)={\dot{Z}}_{c\,0}\geq 0\\ {\ddot{Z}}_{c}\,\left(t_{1}\right)\geq -g \end{array}\right.$$

When the speed of the robot in the horizontal and vertical directions is greater than zero, it will make an oblique throw motion with only the initial speed in the flight phase. It is assumed that the robot makes uniform linear motion in the horizontal direction before jumping, and the velocity of the center of mass in the horizontal and vertical directions is *v_x_* and *v_y_*, respectively. Then, the robot’s flight time is $t=\frac{2\,v_{y}}{g}$, the highest jumping height is $H_{m\,a\,x}=\frac{v_{y}^{2}}{2\,g}$, and the horizontal displacement distance is $S=\frac{2\,v_{x}\,v_{y}}{g}$.

**FIGURE 7:**
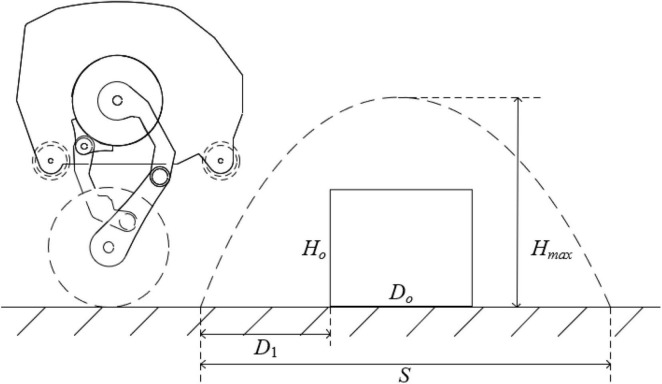
Schematic diagram of the robot over the obstacle.


The obstacle crossing process is shown in Figure 7, it’s required that when the horizontal displacement is *S*_*t*2_ = *D*_1_, the jump height *H*_*t*2_ > *H*_*o*_. When the horizontal displacement is *S*_*t*3_ = *D*_1_ + *D*_*o*_, the jump height *H*_*t*3_ > *H*_*o*_. Then, the conditions under which the robot can overcome obstacles are:

(25) $$\left\{ \begin{array}{c} H_{\max}=\frac{V_{y}^{2}}{2\,g}>H_{o}\\ H_{t\,2}>H_{\text{o}}\\ H_{t\,3}>H_{\text{o}}\\ S=\frac{2\,V_{x}\,V_{y}}{g}>D_{1}+D_{o} \end{array}\right.$$

When considering the robot perform jumps has higher request to the speed and acceleration, vertical direction of the robot center of mass in accordance with the planned five times polynomial interpolation method, the initial and the end of a robot position, velocity and acceleration, respectively, *y*_*init*_, *v*_*init*_, *a*_*init*_ and *y*_*end*_, *v*_*end*_, *a*_*end*_, using the method of undetermined coefficients can get desired trajectory:


(26) $$y\,\left(t\right)=a_{0}+a_{1}\,t+a_{2}+t^{2}+a_{3}\,t^{3}+a_{4}\,t^{4}+a_{5}\,t^{5}$$

Among them,


$$\begin{array}{cc} a_{0}=y_{i\,n\,i\,t} & \\ a_{1}=v_{i\,n\,i\,t} & \\ a_{2}=a_{i\,n\,i\,t}/2 & \\ a_{3}=[20y_{e\,n\,d}-20y_{i\,n\,i\,t}-\left(8v_{e\,n\,d}+12v_{i\,n\,i\,t}\right)t_{4}- & \\ \left(3a_{i\,n\,i\,t}-a_{e\,n\,d}\right)t_{4}^{2}]/2t{}_{4}{}^{3} & \\ a_{4}=[30y_{i\,n\,i\,t}-30y_{e\,n\,d}-\left(14v_{e\,n\,d}+16v_{i\,n\,i\,t}\right)t_{4}- & \\ \left(3a_{i\,n\,i\,t}-2a_{e\,n\,d}\right)t_{4}^{2}]/2t{}_{4}{}^{4} & \\ a_{5}=[12y_{e\,n\,d}-12y_{i\,n\,i\,t}-\left(6v_{e\,n\,d}+6v_{i\,n\,i\,t}\right)t_{4}- & \\ \left(a_{i\,n\,i\,t}-a_{e\,n\,d}\right)t_{4}^{2}]/2t{}_{4}{}^{5} & \end{array}$$

## Robot control system

### Balance and speed control

In general, the wheel-leg movement mode of the robot proposed in this paper can be divided into two forms: wheel movement and jumping over obstacles, which are self-balancing mode and jumping mode, respectively. The robot can switch between the two modes to complete the task, and it is necessary to design controllers for the two motion modes, respectively. The balance and speed control of the robot are realized by the Linear Quadratic Regulator (LQR) method. The state equation of the linear time-invariant system is:


(27) $$\dot{x}\,\left(t\right)=A\,x\,\left(t\right)+B\,u\,\left(t\right)$$

For the balance system of the robot, the speed, tilt angle and tilt angle velocity are taken as the state variables, and the state variable is $x=\left[{\dot{x}}_{b},\phi ,\dot{\phi }\right]$. Then the equilibrium system can be expressed as:


(28) $$\left[\begin{array}{c} {\ddot{x}}_{b}\\ \dot{\phi }\\ \ddot{\phi } \end{array}\right]=\left[\begin{array}{ccc} A_{22} & A_{23} & 0\\ 0 & 0 & 1\\ A_{42} & A_{43} & 0 \end{array}\right]\,\left[\begin{array}{c} {\dot{x}}_{b}\\ \phi \\ \dot{\phi } \end{array}\right]+\left[\begin{array}{c} B_{1}\\ 0\\ B_{2} \end{array}\right]\,T_{\phi }$$

If the robot takes ${\dot{x}}_{r\,e\,f}$ as the reference speed and keeps balance, then $\dot{\phi }=0$, $\dot{x}={\dot{x}}_{r\,e\,f}$, the system stable state variable $x_{s}=f\,\left({\dot{x}}_{r\,e\,f}\right)$, the stability control input $u_{s}=g\,\left({\dot{x}}_{r\,e\,f}\right)$, take the new state variable Δ*x* = *x*−*x*_*s*_, the new system input Δ*u* = *T*_ϕ_−*u*_*s*_, then the new system state equation is:


(29) $$\Delta \,x=\left[\begin{array}{ccc} A_{22} & A_{23} & 0\\ 0 & 0 & 1\\ A_{42} & A_{43} & 0 \end{array}\right]\,\Delta \,x+\left[\begin{array}{c} B_{1}\\ 0\\ B_{2} \end{array}\right]\,\Delta \,u$$

The optimal control input is obtained as follows:


(30) $$u=u_{s}-k\,\Delta \,x$$

Where, $f\,\left({\dot{x}}_{r\,e\,f}\right)=\left[\begin{array}{c} {\dot{x}}_{r\,e\,f}\\ \frac{A_{22}\,B_{2}-A_{42}\,B_{1}}{A_{43}\,B_{1}-A_{23}\,B_{2}}\\ 0 \end{array}\right]$, $g\,\left({\dot{x}}_{r\,e\,f}\right)=\frac{A_{42}\,A_{23}-A_{22}\,A_{43}}{A_{43}\,B_{1}-A_{23}\,B_{2}}$.

### Jumping control

In this paper, the jump control of the robot adopts fuzzy Proportion Differentiation (PD) control. According to the dynamic equation of the jump stage obtained in the dynamic analysis:


(31) $$M\,\left(q\right)\,\ddot{q}+\underset{H\,\left(\bar{q},q\right)}{\underbrace{V\,\left(\dot{q},q\right)+G\,\left(q\right)}}=\tau$$

The control scheme for calculating torque is set as:


(32) $$M\,\left(q\right)\,\left({\ddot{q}}_{d}+K_{p}\,e+K_{d}\,\dot{e}\right)+V\,\left(\dot{q},q\right)\,\dot{q}+G\,\left(q\right)=\tau$$

Where, *e* = *q*_*d*_−*q*, $e={\dot{q}}_{d}-\dot{q}$, *q_d_* and *q* are the ideal angle and the actual angle, respectively.

**FIGURE 8:**
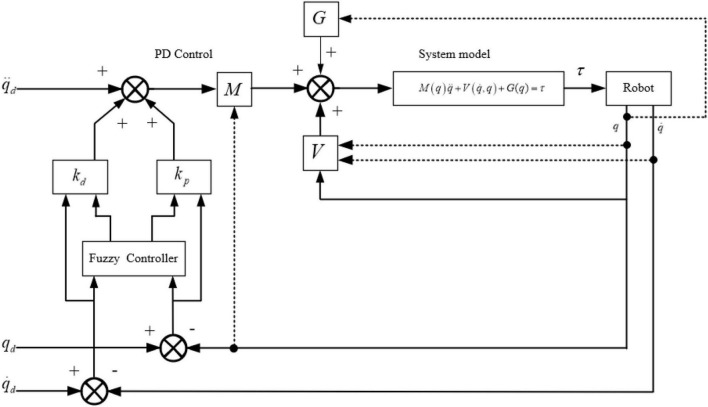
Fuzzy Proportion Differentiation (PD) control block diagram.

The fuzzy PD controller is designed to calculate the torque control, *e* and $\dot{e}$ are taken as the input of the fuzzy controller. Mamdan rule is used for fuzzy inference, and min-max-center of gravity method is used for fuzzy resolution. The output is Δ*K*_*p*_ and Δ*K*_*d*_, let the self-tuning parameters *K*_*p*_ = *K*_*p*0_ + Δ*K*_*p*_, *K*_*d*_ = *K*_*d*0_ + Δ*K*_*d*_. *K*_*p0*_ and *K*_*d0*_ are initial values, KP and KD are final values. The block diagram of the control scheme is shown in Figure 8.

**FIGURE 9:**
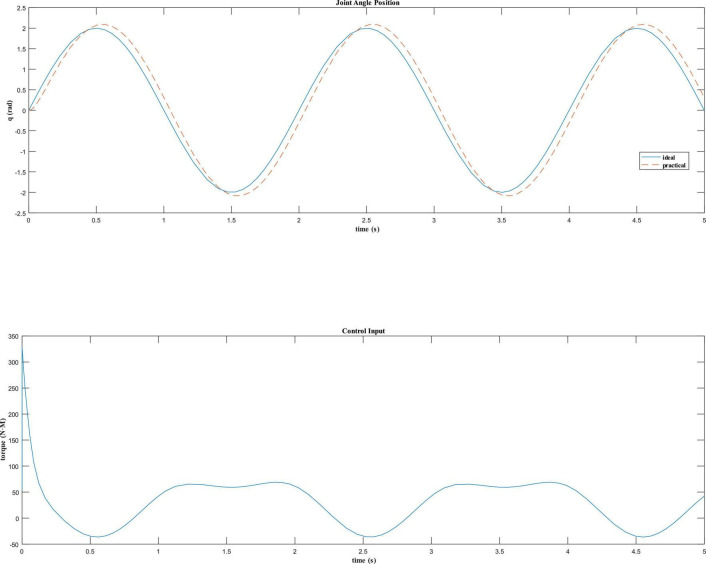
Control effect of fuzzy PD controller.

For the dynamic model of the robot in the take-off phase, assuming that the angle tracking command of the robot hip joint is *q*_*d*_=2sin(π*t*)*rad*, the dynamic equation is written as s-function by the Simulink module in Matlab to test the simulation effect. Fuzzy PD control is used to design the control law, and the initial values of PD parameters are set as *K*_*p*0_=20 and *K*_*d*0_=20. The simulation results are shown in Figure 9.

It can be seen that the joint angle tracking curve of the fuzzy PD controller is close to the expected tracking angle curve in Figure 9, and has a fast response speed, no obvious overshoot, and has a good control effect.

## Simulation and experiment

### Simulation

**FIGURE 10:**
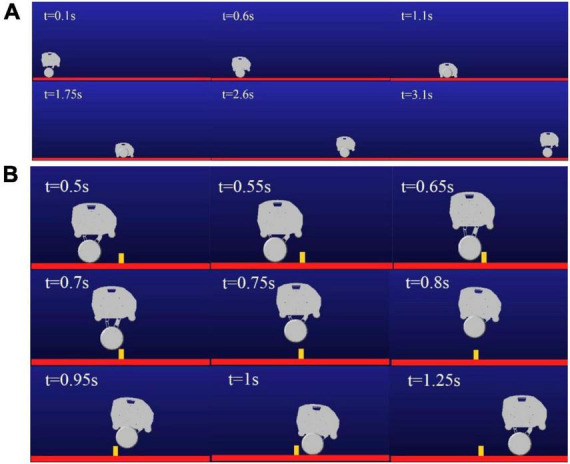
Screenshot of movement process: **(A)** Mode switching process diagram; **(B)** Jump process diagram.

The 3D model of the robot was imported into Adams simulation software, and the co-simulation of the self-balance and obstacle crossing of the robot is carried out by using Simulink module of Matlab. The hip motor rotates at different angles, which can adjust the height of the robot when standing. First, the simulation of the robot shifting from the wheel mode to the wheel-leg mode is carried out. When the hip motors rotate in wheel-leg mode, the robot switches to wheel movement mode, and then reverts to the original posture through motors reversals. The simulation process is shown in Figure 10A. In order to realize the function of jumping over obstacles, the hip motors need to rotate with different steering to complete the contraction and extension of the wheel leg connecting rod, so as to storage and release the energy needed for jumping. Taking the single jump process of the robot as an example, its screenshot is shown in Figure 10B. When the robot moves at a constant speed of 1 m/s, the robot reaches the highest jump height of 0.16 m at 0.8 s. The take-off phase of the robot is 0.5–0.6 s, the flight phase is 0.6–0.95 s, and the landing phase is 0.95–1.2 s.

**FIGURE 11:**
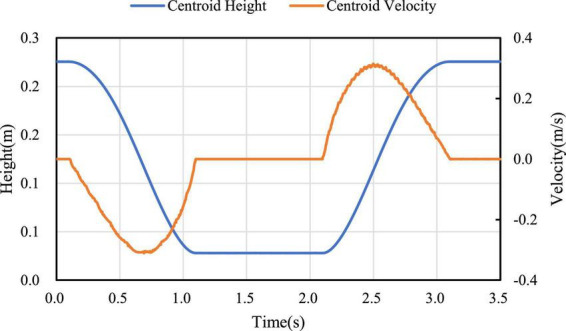
Position and velocity curve of state switching process.

**FIGURE 12:**
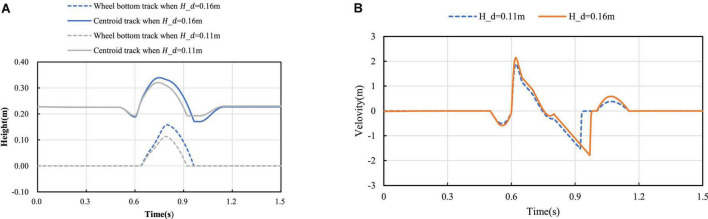
Jump process curve: **(A)** Position curve; **(B)** Vertical velocity curve.

In the process of mode switching, the position and velocity change curve of the robot’s center of mass in the vertical direction are shown in Figure 11. It can be seen that the velocity change curve is smooth and continuous, which can ensure the stable mode switching. According to different expected jumping heights, the jumping trajectories of the robot are different. Jump simulation experiments are conducted for the expected heights *H*_*d*_=0.16*m* and *H*_*d*_=0.11*m*, and the longitudinal trajectories of the bottom of the robot wheel and the robot center of mass are obtained as shown in Figure 12A. It can be seen from Figure 12B, the motion of the robot after take-off is oblique throwing motion, and the planned motion wants to achieve a higher jumping height, so a larger longitudinal velocity is needed at the take-off point to prolong the time of flight. In the take-off phase, the wheel-leg linkage mechanisms successively contract and extend to realize the energy storage and release of the machine mechanisms. When the condition of leaving the ground is reached, the end of the robot’s wheels leave the ground and enter the phase of flight. In the flight phase, the robot system is in the state of momentum conservation, so the method of contracting the wheel-legs to increase the jumping height causes the overall centroid velocity to fluctuate. In order to reduce the impact of landing, a method is used to keep the mechanism retraction until the wheels touch the ground. After the robot lands, the wheel-legs extend back to the position before the jump.

**FIGURE 13:**
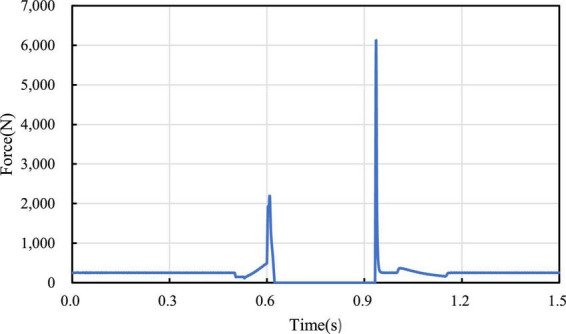
Ground support reaction force variation curve.

In Figure 13, the curve of ground support reaction force on the wheel in the process of jumping is measured, and it’s found that the support reaction force gradually increases in the take-off phase, reaches the maximum value at 0.6 s at the end of wheel-leg contraction, and decreases rapidly at the moment of take-off. When the wheels leave the ground, the support reaction force is 0. At the moment of landing, the support reaction force will change due to impact. The change of the support reaction force corresponds to the height above the ground in each stage of the robot jumping process, which verifies the feasibility of the jumping.

**FIGURE 14:**
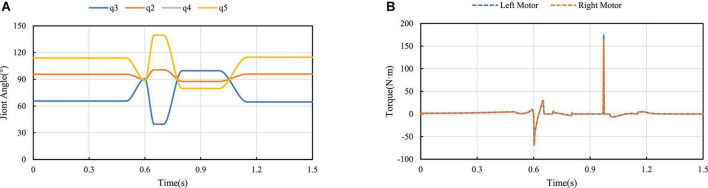
Curve change of jump process: **(A)** Curve of joint angle change; **(B)** Output torque diagram of motors.

Figure 14A shows the angle variation of each joint when the jump height is 0.16 m. Since the wheel-leg of the robot is a parallelogram linkage mechanism, *q*_4_ = *q*_5_. Among them, the angles *q_2_*, *q_3_*, *q_4_*, and *q_5_* represent are the angle between Connecting rod 3 and Connecting rod 1, the angle between Connecting rod 3 and Connecting rod 2 at the hinge point, The angle between Connecting rod 2 and the motor rotation center and the Connecting rod 2 hinge, and the angle between Connecting rod 2 and Connecting rod 3. Robot hip motor torque curves is shown in Figure 14B, it can be seen two hip motors’ output torques are basically consistent, the motors’ torques of the wheel-legs contraction in the take-off phase are shown in the curves from 0.5 to 0.6 s, which are the torques curves of the wheel-legs extension from 0.6 to 0.65 s, and the torque generated near 0.97 s is generated when the robot lands and touches the ground.

**FIGURE 15:**
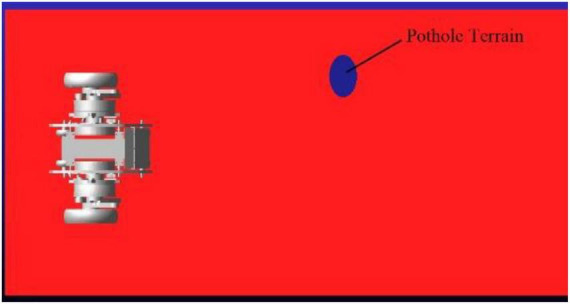
Schematic diagram of pits.

**FIGURE 16:**
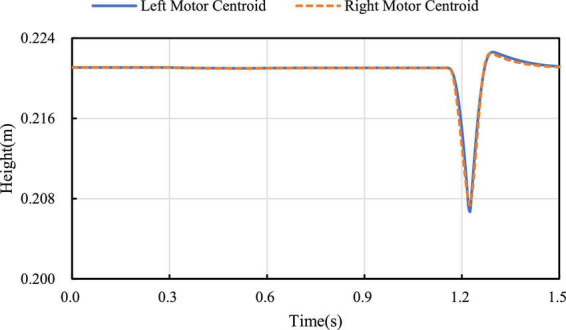
Left and right motor location diagram.

When the robot passes over the pavement with pothole (major diameter: 15 cm, minor diameter: 10 cm, depth: 5 cm) as shown in Figure 15, the robot is required to keep moving smoothly. When the left leg of the robot passes over the pothole, the linkage mechanism of the right leg contracts to keep the axes of the motor rotation of the hip joint of the two legs coincide as much as possible. Figure 16 shows the height of the rotation axes of the left and right wheel-leg hip motors in this process. It can be seen that the axes of the two motors basically coincide. At time of 1.2 s, the deviation of the axis of the two legs is relatively large, but as a whole, the deviation is small, only about 2 mm. The robot can smoothly pass through the pothole terrain.

**FIGURE 17:**
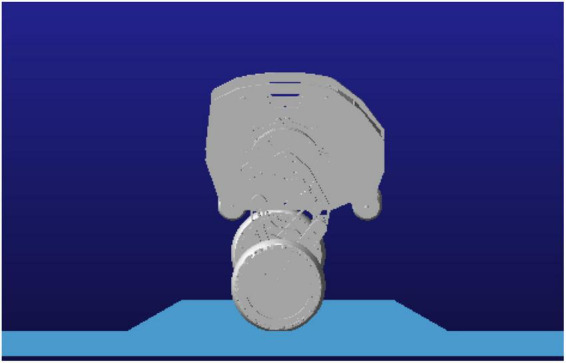
Obstacle crossing of wheel-leg without adaptive contraction.

**FIGURE 18:**
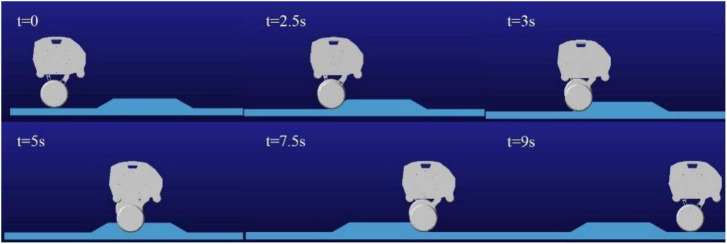
Process diagram of single-leg obstacle crossing.

The following simulation experiments are done for the robot to cross the slope obstacle with one leg. The obstacle set by the simulation is about 6.5 cm in height and 65 cm in length. When the robot crosses the obstacle without adaptive contraction wheel-legs, the side view of the obstacle crossing process of the robot is shown in Figure 17. In this case, crossing the obstacle may cause damage to the connecting rod structure of the robot wheel-leg or the motors of the hip joints. Therefore, when the vertical height of the contact between the left and right wheels and the ground is different, the wheel-leg linkage mechanism with relatively high vertical position of the contact point needs to be properly contracted to ensure the stability of the robot body. For this purpose, the one-leg obstacle crossing simulation experiment was carried out, and the whole process of obstacle crossing was captured, as shown in Figure 18.

**FIGURE 19:**
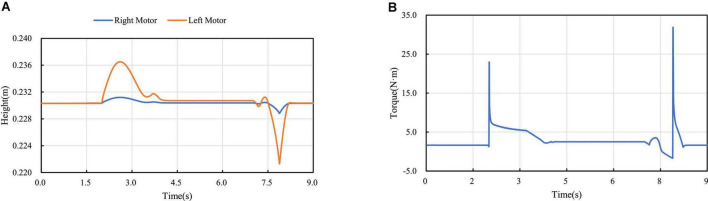
Crossing the obstacle with one leg: **(A)** Diagram of motors’ locations; **(B)** Diagram of motors’ torques.

Figure 19A shows the height variation curve of the rotation axis of the left and right hip joint motors in the vertical direction. It can be seen from the figure that the left leg of the robot starts to cross the obstacle when it contacts the obstacle at about 2 s, and the robot starts to leave the obstacle at about 7.15 s. In the process of obstacle crossing, the highest position deviation of the motor axis of the hip joint of the two legs is generated at 7.9 s, and the maximum deviation between the motor position of the left leg and the motor position of the right leg is about 6.6 mm. It can be seen that in the whole process of obstacle crossing, the position height deviation of the two hip motors is low, and the performance of obstacle crossing is better. And because of gravity, the time for going up and down is different, and the time for going down is less than that for going up. Figure 19B is the robot’s left leg hip motor torque figure, can be seen from the figure when the robot starts uphill because access to the obstacles, will jump a torque value, and in the process of uphill, with the contraction of the wheel-leg, it gradually attenuates to the torque value of maintaining the motor position locking, and also produces a sudden torque when downhill.

### Experimental verification

**FIGURE 20:**
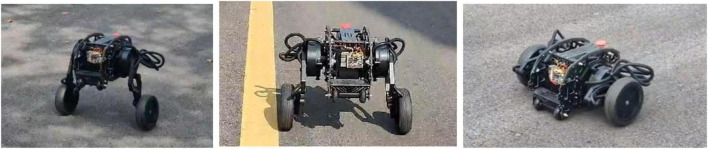
Schematic diagram of different motion states.

On the basis of simulation, in order to verify the feasibility of jumping over obstacles designed in this paper and the correctness of the simulation results, real robot tests are carried out. The experiments are based on the DDT robot platform of Direct Drive Technology Ltd. The total mass of the robot is about 30 kg, the height of the robot in the wheeled mode is about 21 cm, the length of the robot is about 35 cm, and the width is about 53 cm. The height of the robot can be adjusted under the wheel-leg mode. The robot moves in wheel mode and wheel-leg mode at different height of center of mass on flat ground, the motion posture is shown in Figure 20.

**FIGURE 21:**
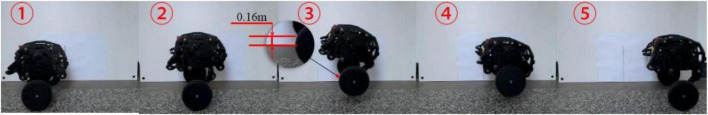
Diagram of jump process.

The real tests are carried out in different terrain, first of all, the jump height and jump feasibility will be analyzed and verified under flat ground. Control the robot to jump and compare the jump height with the scale of the preset whiteboard (see Figure 21). The energy needed for the robot to jump is stored by the contraction of the leg mechanisms, the energy release is completed by the extension of the leg mechanism and the jump is carried out, the wheel-legs are contracted in the flight phase to improve robot’s height off the ground, and the purpose of jumping over obstacles is realized. After comparing with the scale of the white board, it is found that when the robot jumps at a low speed, the jump height can be 0.16 m. The experimental results are basically consistent with the simulation results, which verifies the feasibility of the jump action of the wheel-legged robot designed in this paper.

**FIGURE 22:**
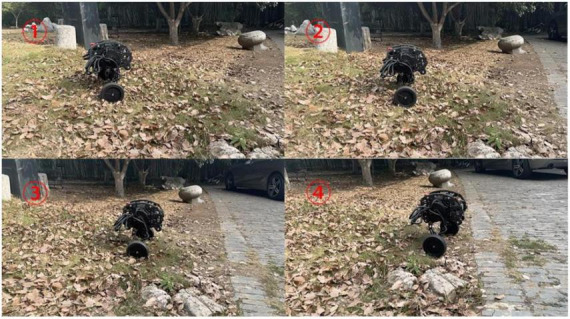
Moving over potholed ground.

The adaptive contraction of the wheel leg is actually tested on the pothole ground. In Figure 16, it can be seen that in the simulation experiment of pothole terrain, the robot passes through the pothole in about 1.2 s, corresponding to the robot state shown in serial number ① and serial number ② of Figure 22. At this time, the robot walks on the road with potholes. The axes of the two hip motors are always in the same vertical position by contracting the wheel-leg which is at a higher position, it ensures the stability of the robot when walking on the pothole ground.

**FIGURE 23:**
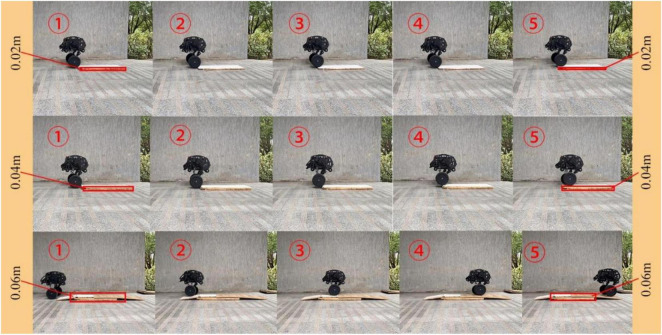
Comparison of different height of obstacle crossing.

For the test of single-leg obstacle crossing, a single block, two blocks and three blocks of wood are set in front of the right leg as obstacles. The height of a single plank is 2 cm, the height of the corresponding obstacles is 2, 4, and 6 cm, respectively, and the radius of the wheel is 9.5 cm. The obstacle crossing process is shown in Figure 23. The process from contacting the obstacle to adaptively adjusting the expansion of the wheel-leg is shown. It can be seen that the degree of contraction of the wheel-leg is different when the height of the obstacle is different. The robot can easily cross the obstacles with height lower than the radius of its own wheels, and keep moving smoothly through obstacles by adjusting the wheel-leg adaptively.

**FIGURE 24:**
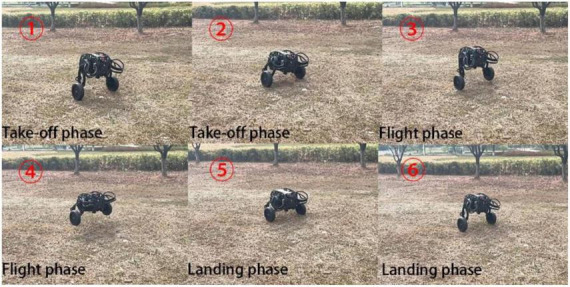
Jumping process on uneven ground.

In addition to jumping on the flat and open ground, jumping over obstacles on the uneven ground is an important embodiment of the robot’s ability to jump over obstacles. The jumping ability was further tested on the uneven grass outside, and the test results in Figure 24 shows that the robot still had good jumping ability on the uneven ground.

## Conclusion

In this paper, a bipedal wheel-legged robot with parallel four-bar linkage wheel-leg structure is proposed. The kinematics and dynamics of the robot are analyzed, and LQR controller and fuzzy PD controller are designed for balance and jump, respectively. According to the output torque curve and hip joint angle tracking curve obtained by Simulink simulation experiment, it can be seen that it has a good control effect. In view of the different ground conditions that the robot may encounter in the complex terrain environment, Adams and Simulink are used to simulate the robot’s obstacle crossing, respectively, for the pothole road surface, the obstacle height is higher than the wheel radius and the obstacle crossing with one leg. Under the gait strategy of adaptive wheel-leg contraction, the error of each simulation data of the robot is small and the output torque is within the effective output range of the motor, which can ensure the smooth obstacle crossing. In the jump simulation, when the expected jump height is 0.11 and 0.16 m, the vertical velocity of the former is lower than that of the latter. To increase the height of obstacle crossing, the vertical velocity of the take-off should be increased. The gait strategies used in each simulation experiment are verified in real scene test, and the robot can smoothly cross the obstacles, which verifies the feasibility of the jumping and control method.

## Data availability statement

The original contributions presented in this study are included in the article/Supplementary material, further inquiries can be directed to the corresponding author.

## Author contributions

TG: methodology, project administration, and writing—review and editing. JL: simulation, analysis, experiments, and writing—original draft preparation. HL: control system design. YZ: conceptualization, design, and mechanical modeling. WC, XX, and MW: supervision and review. ZW: funding acquisition. All authors read and agreed to the published version of the manuscript.
